# Use of Light Stimuli as a Postharvest Technology for Cut Flowers

**DOI:** 10.3389/fpls.2020.573490

**Published:** 2020-12-21

**Authors:** Takanori Horibe

**Affiliations:** College of Bioscience and Biotechnology, Chubu University, Kasugai, Japan

**Keywords:** circadian rhythm, flower opening, flower quality, petal growth, photoperiod

## Introduction

Cut flowers are a unique aspect of the flora industry, and they are globally recognized as one of the most commercially produced ornamentals. Thus, it is necessary to increase their quality for consumers' satisfaction over a long period. The quality of a cut flower depends on its external characteristics such as color, length, volume, freshness, and fragrance as well as its perishability, which is determined by the duration of these external characteristics. Recent researches have shown that external light emission significantly affects several aspects of cut flower traits, such as; the vase life, petal color, and volatile compounds emission. Clarifying the relationship between these flower traits and light stimuli, which can increase the understanding of the mechanisms of these traits, can help to improve the quality of cut flower through light environmental control. This study proposes that the external light stimuli is an effective tool for improving the quality of cut flowers, and also highlights the several issues related to this proposal.

## Postharvest Technology for Cut Flowers

The vase life is a critical factor in determining the market value of cut flowers. However, since it takes some days to sell cut flowers from farmers via retailers to general consumers, the quality of the cut flowers could depreciate during this process. For this reason, most cut flowers lose their ornamental value within 1 week after being purchased by consumers, to the dissatisfaction of the customers. The quality of cut flower and its vase life are affected by several factors. These include the aging, detachment, and withering of tissues by ethylene generation, deficiency in saccharides that act as osmoregulators and respiratory substrates, obstruction of vessels due to bacterial propagation, and generation of air embolism in stem (poor water absorption) (Burdett, 1970; Durkin, 1979; De Stigter, 1980; Reid et al., 1989; van Doorn, 1997; Macnish et al., 2010).

Continuous efforts have been made to improve the quality of cut flowers and prolong their vase life, which has led to the development of numerous agents to preserve flower quality (van Doorn and Woltering, 2008). Ethylene inhibitors, such as silver thiosulfate complex (STS) and 1-methylcyclopropene (1-MCP) are widely used as quality retention agent because ethylene is a major factor in the aging of many cut flowers (Veen, 1979; Mor et al., 1983; Ichimura et al., 2002). Quality preservatives include ethylene production/action inhibitors, sugars, antibacterial agents, plant growth regulators, surfactants, and inorganic salts (van Doorn and Woltering, 2008; Ichimura, 2018). Commercially available preservatives consist of a mixture of these various agents, but the detailed composition of the ingredients is usually concealed. However, some quality preservatives, such as STS causes environmental damage, and many countries prohibit using them in order to sustain environmental protection (Tacuri et al., 2016). The negative effect of STS in the environment is mainly due to the residual effects of the inherent metal it deposits into the soil and groundwater for long period and its potential migration to drinking water systems (Tacuri et al., 2016).

It has also been found that high temperatures during transport or storage significantly reduce the vase life and quality of cut flowers, and make ethylene-sensitive species more susceptible to ethylene (Halevy and Mayak, 1973; Rudnicki et al., 1991; Leonard et al., 2011). For example, a previous study showed that exposing flowers to 20°C for 8 h early in the marketing chain accelerated the opening of the *Aster* spp., *Chrysanthemum* spp., *Dianthus* spp., and *Gypsophila* spp. compared to continuous exposure of 8°C (van Gorsel and Ravesloot, 1994). The effect of the higher temperature became more apparent later in the marketing chain. However, the mean interruption temperatures showed that 1 day delay in the marketing chain resulted in 1 day (*Aster* spp. and *Gypsophila* spp.) to 3 days (*Dianthus* spp. and *Chrysanthemum* spp.) decrease in vase life (van Gorsel and Ravesloot, 1994). Thus, there has been a considerable emphasis and discussion within the floral industry regarding the need of cold-chain, which is a type of supply chain that is temperature-controlled from the point of production, to the transportation stages, storage, distribution processes and finally delivery to the end-user (van der Hulst, 2004; Reid and Jiang, 2005; Leonard et al., 2011). Although automatic machines have been invented to improve the efficiency of the fresh flower logistics, a large part of the logistics process is handled manually because various types of fresh flowers are difficult to control by only machines (Babalola and Sundarakani, 2011). Thus, there is often a lack of cold-chain during logistics, leading to the exposition of flowers to high and fluctuating temperatures. Furthermore, establishing perfect cold-chain needs a lot of initial investment and running costs (e.g., fuel and energy).

Thus, it is desirable to introduce a new technology that is inexpensive, of low environmental load, and can improve the quality of cut flowers.

## Use of Light for Improving Cut Flower Quality

Light acts as an environmental signal and also drives photosynthesis in plants. Plants respond to the intensity, wavelength, duration, and direction of light (Zheng and Labeke, 2018), and the use of various light sources such as light-emitting diodes (LED) has been experimented in agricultural science. For example, long-day treatment (night interruption) using incandescent and fluorescent lamps during the short day, which effectively accelerate the flowering of long-day plants and allowed earlier marketing or seed production, has been applied to numerous long-day plants in Japan, including *Ammi majus* L. (Tsuchiya, 1991), *Bupleurum griffithii* L. (Hara and Katsutani, 1993), *Campanula* spp. (Moe et al., 1991).

Recent researches have shown that the light environment also affects diverse aspects of cut flower qualities. For example, on the effects of light environment on flower opening in cut rose (*Rosa hybrida* L.) revealed that red light suppressed flower opening and wilting, resulting in longer vase life compared with other treatments; blue light, white fluorescent light, and constant darkness condition (Horibe et al., 2020). On the other hand, the red light was shown to promote flower opening, but its effect was reversed by subsequent exposure to far-red light in *Ipomoea nil* L. (Kaihara and Takimoto, 1980, 1981). It has also been observed that high light intensity extends vase life in *Anthurium andraeanum* H, *Chrysanthemum* spp., *Delphinium* spp. and *Dianthus caryophyllus* L. (Pun and Ichimura, 2003; Evelyn et al., 2020). Flower color is also an important factor affecting cut flower quality, and light spectra such as ultraviolet (UV) and blue light is involved in anthocyanin biosynthesis of flowers. For example, UV and blue light irradiation increase anthocyanin biosynthesis in petals of cherry blossom (*Prunus* × *yedoensis* “Somei-yoshino”), *Gerbera hybrida* H*., Malus domestica* L. and *Rosa hybrida* L. (Maekawa et al., 1980; Dong et al., 1998; Meng et al., 2004; An et al., 2020). Furthermore, light stimuli are closely related to floral scent emission. Studies have shown that several scent components are under the control of the circadian clock, and plants can quickly entrain their scent emission to the new light/dark cycles after shifting the photoperiod (Hendel-Rahmanim et al., 2007; Fenske and Imaizumi, 2016). Blue light has also been shown to suppress the development of blue mold (*Penicillium italicum*) in *Citrus unshiu* M. and gray mold (*Botrytis cinerea*) in *Solanum lycopersicum* L., and at least, these effects could be partially related with the enhanced proline accumulation and antioxidative processes (Kim et al., 2013; Yamaga et al., 2015). Since gray mold caused by *Botrytis cinerea* is one of the most important diseases of several ornamental flowers including *Chrysanthemum* spp., *Begonia* spp., *Dahlia* spp., *Geranium* spp., *Gerbera* spp., *Tulipa* spp*., Rosa hybrida* L., and *Viola* spp. (Marrois et al., 1988; Terry and Joyce, 2004; Araújo et al., 2015), blue light irradiation may become an effective way to suppress pathogenic development in cut flowers. Furthermore, several studies have revealed that flowers are the site of photoperception and synchronize their growth to photoperiods (Saito and Yamaki, 1967; Kaihara and Takimoto, 1980, 1981). In *Calendula arvensis* L., the opening and closing rhythm of flowers followed the light/dark cycle to which flowers had been exposed when the leaves were subjected to a different cycle from the flowers (van Doorn and van Meeteren, 2003). Horibe and Yamada (2014) reported that even a petal removed from a rose flower showed rhythmic growth when exposed to a 12 h light/12 h dark photoperiod, indicating that petals could perceive light and synchronize their growth to the photoperiod. These results have shown that light affects various important traits of cut flower quality including vase life, petal color, fragrance, and perishability. Therefore, we think light environment control can be a remarkable quality preservation technique, despite that its commercial use is still inadequate compared to existing technologies such as the use of preservatives and cold-chain.

Then, what kind of mechanisms underlie the responses of cut flowers to light stimuli? Cut flower reactions toward light stimuli including rhythmic growth, volatile compound emission, pigment synthesis, and disease resistance should be regulated through the interaction between plant photoreceptors sensitive to a particular wavelength and their downstream signaling pathways. In cut carnation (*Dianthus caryophyllus* L.), blue light delayed the petal senescence through down-regulation of ethylene biosynthetic genes and up-regulation of antioxidant enzymes (Aalifar et al., 2020a,b). It is well-recognized that adverse water relation caused by water loss from stomata and cuticular can lead to problems in flower opening, such as premature petal wilting and bending of pedicel, resulting in short vase life (van Meeteren and Aliniaeifard, 2016; Aliniaeifard and van Meeteren, 2018). In these experiments, blue light also promoted stomata opening and water uptake (Aalifar et al., 2020a,b). Elevated water uptake and higher transpiration rates are reported to be beneficial for cut flowers placed in water (Elibox and Umaharan, 2010; Lu et al., 2010). Aalifar et al. (2020a,b) reported that blue light might increase water transport efficiency by increasing transpiration rate and water uptake. However, petal growth and vase life of cut roses changed when exposed to different light environments even without leaves (Horibe et al., 2020), indicating that responses of cut flowers to light stimuli cannot be explained only by the function of leaves. In addition, blue light resulted in shorter vase life in cut anthurium (*Anthurium andraeanum*) under cold storage and the negative effect of blue light was attributed to its effect on increased water loss and oxidative stress (Aliniaeifard et al., 2020). There are still many unsolved questions between light stimuli and flower responses. What is the source of discrepancy in the reports on the effects of light on cut flower postharvest quality? Why different cut flowers respond differently to the light quality or intensity? Can light environment control prevent postharvest disease outbreak and infections? However, most of the details are still unknown. Further research is needed to explain the above phenomenon, and these would be an interesting topic from a viewpoint of plant physiology.

Challenges for the use of light environment control as postharvest technology are its initial and running costs. However, with the use of LED, which has low power consumption, low heat generation, and adjustable wavelength, the costs would be minimized compared with other light sources (Hong et al., 2011). Initial and running costs would not be high when compared to the establishment of cold-chains, since light environmental control can be possible with only lighting devices. Besides, it is relatively easy to use and does not require much labor. Furthermore, it can be used in combination with the conventional quality retention technology such as use of quality preservatives and temperature control (cold-chain). Therefore, we believe that the light environment control including wavelength and photoperiod can be a promising tool for improving the quality of cut flowers during transportation and storage.

## Final Remarks

As indicated above, light environment control could be a powerful tool for improving postharvest quality of ornamental cut flowers. Recently, there is an urgent need for sustainability and food security that should lead to advancement in horticulture market such as increased production of fruit and flowers. In this context, efforts have been made to reduce the use of inorganic pesticides and natural substance to prevent the harmful effects of chemical compounds that threaten both the environment and human health. Compared to the existing technology, such as the application of quality retention agents to vase water, light environment control has little negative effect on environment and is well-suited with an environmentally-friendly market. Further research is needed to determine the reaction of cut flowers toward light among species and cultivars, and investigate how light stimuli affect several features of cut flowers. To apply light environment control into the floral industry for the improvement of several cut flower qualities, more practical attention should be given to this strategy.

## Author Contributions

TH wrote the article.

## Conflict of Interest

The author declares that the research was conducted in the absence of any commercial or financial relationships that could be construed as a potential conflict of interest.
